# Influencing Factors of Void closure in Skew-Rolled Steel Balls Based on the Floating-Pressure Method

**DOI:** 10.3390/ma12091391

**Published:** 2019-04-29

**Authors:** Chang Shu, Jitai Wang, Xuedao Shu, Duanyang Tian

**Affiliations:** 1 The William States Lee College of Engineering, University of North Carolina at Charlotte, Charlotte, NC 28223, USA; cshu@uncc.edu; 2 Faculty of Mechanical Engineering and Mechanics, Ningbo University, Ningbo 315211, China; Lizixuan@nbu.edu.cn (J.W.); tianduanyang@whut.edu.cn (D.T.); 3 Zhejiang Province Key Lab of Rolling Part Technology, Ningbo University, Ningbo 315211, China

**Keywords:** FPM, void closure, skew rolling, steel ball, void

## Abstract

Due to the instable conditions caused by the wear of rollers, macro voids inevitably occur in skew rolling steel balls. Macro voids in rolled balls greatly weakens the mechanical properties, resulting in the scrapping of about 23% of all skew rolling balls. This paper adopts the floating-pressure method (FPM) to eliminate macro voids in rolled steel balls, and mainly focuses on the investigation of the influencing factor void closure in skew-rolled balls. The research contents are listed as follows: Firstly, the mechanical model of FPM eliminating void in rolled steel balls is established, and the theoretical relationship between influencing factors of void closure is obtained. Then, the metal flow behaviors, the stress distribution and the effect of process parameters on void closure are revealed by numerical analysis. Subsequently, based on the uniform design method, the prediction equation of the required temperature and air pressure for compacting various inferior rolled balls with different diameter by FPM is deduced. Finally, the FPM experiment is carried out to verify the results of numerical analysis. The research results provide theoretical guidance for eliminating macro voids in skew-rolled steel balls.

## 1. Introduction

**Figure 1:**
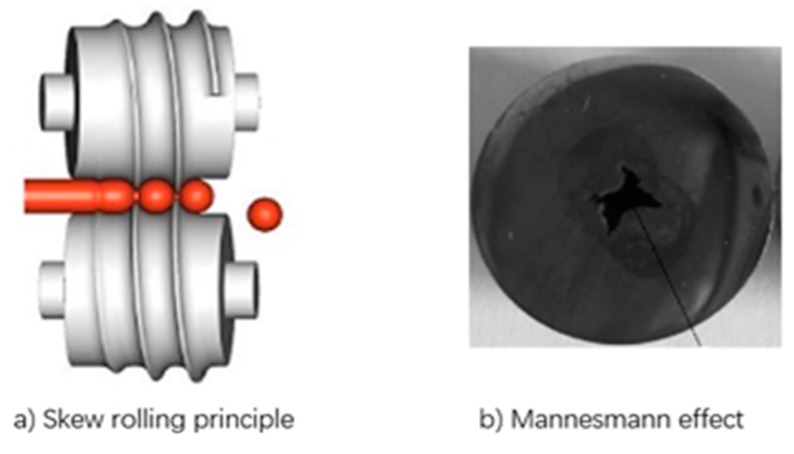
Scheme diagram of skew rolling steel balls and Mannesmann effect. (**a**) Skew rolling principle; (**b**) Mannesmann effect.

The skew rolling process is widely applied in the near net forming of bearing steel balls due to its several advantages such as high working efficiency, high material utilization rate, low cost and low forming force, and the fine machining of skew rolling must be arranged to guarantee the final dimension of bearing steel balls. As shown in Figure 1a, skew rolling is a complicated rolling process, in which a bar billet rotates along its own axis and axially translates forward under the extrusions of two or three rollers with a volute groove so as to form the desired ball. However, the process of skew rolling ball becomes more instable with the wear of rollers, inevitably giving rise to the appearance of plenty of inferior products with macro void, which is so-called the Mannesmann effect, as shown in Figure 1b. That is to say, we can only produce a high-quality bearing ball by a skew rolling process with no wear on forming dies. According to the data from one local skew rolling factory, about 23% of all skew-rolled balls are inferior products with macro voids, and macro void inside a steel ball greatly weakens the mechanical properties of the ball, and shortens the service life of a ball as well. With regard to those inferior balls with macro voids, macro voids inside rolled steel balls cannot be eliminated by next fine machining, and thus, it would be faced with scrap disposal, resulting in a great waste of material. A high degree of waste material and resources is one of the most notable social problems in the 21st century of energy shortage, and how to improve the utilization of material and energy becomes the focus of concern for many enterprises and scholars.

By now, lots of studies have been conducted on evaluating and improving the internal quality of skew rolling steel balls. Cao et al. and Du et al., illustrated that macro voids inside skew-rolled ball are caused by the joint efforts of the cyclic alternating shear stress, the lateral tension stress and the mean stress, and explored that the forming stability of skew rolling ball can be enhanced, to some extent, by optimizing the stress state inside steel balls [1,2]. By means of metallographic experiments and scanning electron microscopy, Lei et al., declared that macro void in the core of hot-rolled steel balls belongs to the metallurgical defect, and they suggested using non-defective raw materials may impede the occurrence of macro voids in rolled steel balls to some extent [3]. In addition, some scholars studied the deformation mechanism in skew rolling steel balls by analyzing the metal flow behaviors, stress states and microstructure evolution [4,5,6,7,8,9,10,11,12,13], which could provide theoretical guidance for the defect control in skew rolling steel balls. The above references mainly focus on the analysis and control of defects during the process of skew rolling steel balls, but the radical problem of macro voids induced by roller wear cannot be avoided. However, as the shape of skew-rolled ball would sharply change when using the traditional forging technology to compact macro voids inside rolled ball, there are not any reports on repairing the defects inside rolled steel ball. Therefore, it is valuable to find a novel process to eliminate macro void inside skew-rolled steel balls on the premise of ensuring the dimension accuracy.

**Figure 2:**
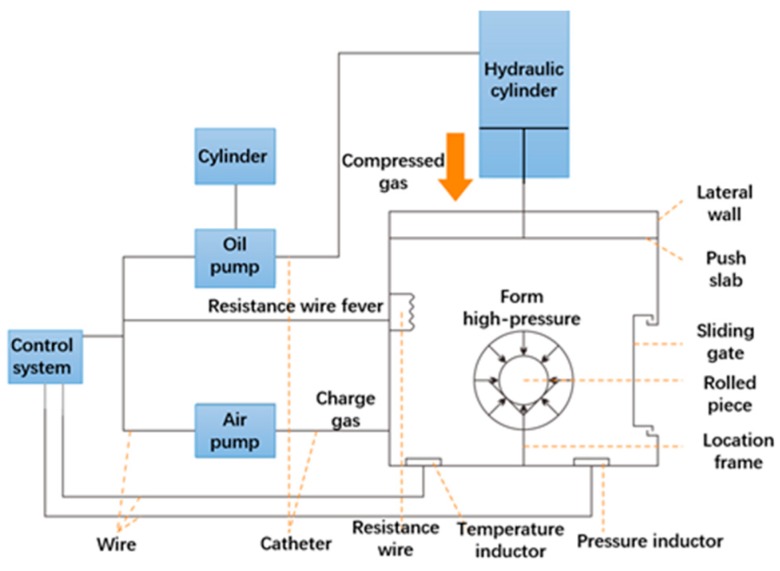
The principle of compacting voids in skew rolling ball by the FPM.

Inspired by an ideal that the shape would not obviously variate in the case that the surface of inferior rolled steel ball is subjected to the action of equal loads, the floating-pressure method (FPM) has been proposed by co-authors, of which the scheme diagram exhibited in Figure 2 can be described as follows [14,15,16]: (1) Several inferior rolled balls are firstly placed in the FPM device, and then the device is strictly sealed. (2) Oil pump, air pump and resistance wire begin to operate under the control of the Computer Numerical Control (CNC) system, which could provide the circumstances of high temperature and air pressure. (3) Rolled steel balls with macro voids need to be subjected to the actions of high temperature and air pressure for a long time so as to ensure the complete elimination of macro voids. This paper aims to investigate the influencing factors of void closure inside rolled ball and establish the prediction model of the required temperature and air pressure. The remaining frame of this work is arranged as follows: In Section 2, the mechanical model of void closure inside rolled steel ball based on FPM is established, and the theoretical relationships between influencing factors of void are obtained. In Section 3, the material constitutive models are firstly presented, and then the Finite Element (FE) model of eliminating void inside rolled steel balls by FPM is established. In Section 4, the metal flow behaviors, the effective stress distribution and the influencing factors of microstructure in rolled steel ball are discussed. In Section 5, based on the uniform test design method, the prediction equation of the required temperature and air pressure for various steel balls with different diameter are deduced. In Section 6, the FPM experiment is carried out to verify the results of numerical analysis. In Section 7, we draw some primary conclusions relating to future work.

## 2. Mechanical Model of Void Closure in Rolled Steel Balls

**Figure 3:**
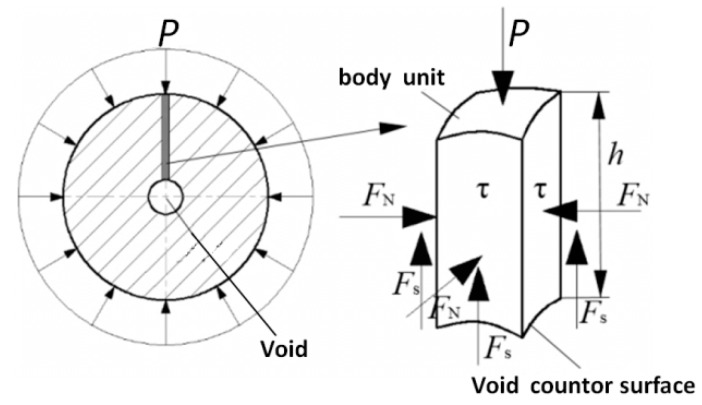
The mechanical model of void closure based on the FPM.

Selecting the steel ball with spherical void in the core as the research object, cut the cross section at the center of the ball as shown in Figure 3, and take a unit body along the contour of the void (the height of the body unit is “h”, the length of the bottom side is “1”). Since the closure of the void is mainly caused by the flow of the outer metal, the force analysis of the body unit is performed in the vertical direction.

The body unit is mainly subjected to the air pressure *P*, the shear stress *τ*, and the force *F_S_* of the adjacent body unit in the vertical direction, and an equilibrium equation as shown in the Equation (1) is established.

(1) $$4\tau \cdot h=P_{4}-F_{S}$$

Since the circumferential direction of the rolled piece is highly symmetrical, the vertical force component *F_N_* of adjacent unit of the body unit is proportional to the air pressure *P*, and *F_N_* can be calculated according to the Equation (2), where *h* is the distance from the void center to the circumferential surface of the rolled piece. Since the void is usually located in the center of the ball and its size is much smaller than the diameter of the rolled piece, *h* is regarded as the radius *r* of the rolled piece; *J* is the material and the ball shape-related conversion factor.

(2) $$F_{S}=J\cdot P\cdot h$$

Substituting Equation (2) into Equation (1), we can obtain:

(3) $$\tau =P\left(\frac{1}{4r}-J\right)$$

When *τ* is greater than the yield stress σ_s_ of the material, metal rheology appears and drives void deformation. The shear stress is proportional with the air pressure and negatively correlated with the diameter of the rolled piece. Therefore, it can conclude that the larger the diameter of the rolled piece is, the greater air pressure will be required to compact its internal void.

The process of compacting void inside skew-rolled ball by FPM has two stages [16]: (1) void shrinks to several micro needle-like gaps (also called void closure); (2) the welding of several micro needle-like gaps (also called interface solid welding). Some scholars devoted a lot of efforts to investigate the solid bonding of macro void in metal material. Zhu established the mesoscopic damage model of large ellipsoidal part with pretty small ellipsoidal void based on the upper bound calculation method [17]. Cui et al. obtained the conditions of void closure in cylinder by the compression tests of cylindrical rod [18]. Zhang developed a program of predicting the solid bonding state of void in forming the large forgings, and successfully penetrated the program into MSC Marc 2018 software [19]. Saby et al., summarized typical void closure criteria for hot metal forming [20]. In order to heal all microcracks in rolled ball to improve the mechanical properties, the free diffusion of high-temperature atoms and dynamic recrystallization are expected to occur. In addition, the compressive stress and temperature should reach a certain level. Due to the relatively uniform force of the metal near the crack, the compressive stress can be replaced by the equivalent stress. Theoretically, as long as the temperature and equivalent stress at the crack reach a certain level, the crack can be healed. According to the relevant literature [21], the conditions for void healing are obtained as shown in Equation (4). That is to say, when the equivalent stress near the void is greater than the yield stress of the material, the void can be welded.

(4) $$\bar{\sigma }\geq \sigma_{s}$$

## 3. Material and FE Model

### 3.1. Material Model 

**Figure 4:**
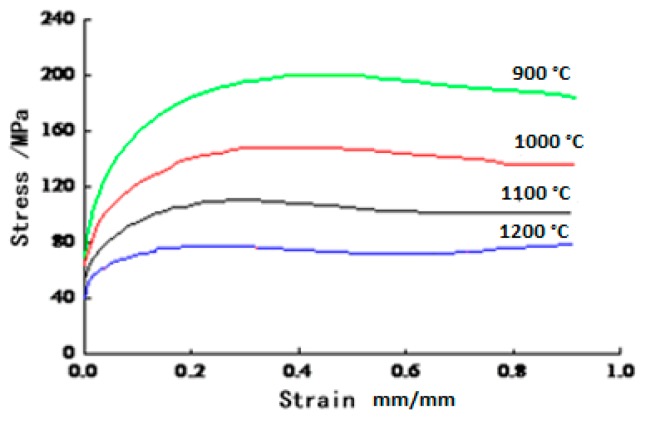
True stress-strain curves of 42CrMo at different temperatures at the strain rate of 0.01 s^−1^.

42CrMo is selected as the materials of the simulation model [22,23,24,25]. At 0.01 s^−1^ strain rate, the true stress-strain curves of 42CrMo at different temperatures are shown in Figure 4, and the curve is subjected to multivariate nonlinear fitting. The constitutive equation of the 42CrMo is shown in Equation (5).

(5) $$\dot{\varepsilon }=1.34\times 10^{18}{\left[\sinh(8.198\times 10^{-3}\sigma )\right]}^{8.1434}\times \exp\left(-\frac{4.6334\times 10^{5}}{RT}\right)$$

In the formula, R is the gas constant, which is 8.31 J/(K·mol); T is the deformation temperature.

The critical strain of dynamic recrystallization can be estimated from the peak strain of the stress-strain curve. Dynamic recrystallization (DRX) occurs before the stress reaches the peak. When the stress reaches the peak stress, the corresponding strain can reflect the deformation state of the material.

The dynamic recrystallization activation criteria can be described as:

(6) $$\varepsilon_{p}=7.28\times 10^{-4}d_{0}^{0.31}{\left[\dot{\varepsilon }\exp^{\frac{247000}{RT}}\right]}^{0.21}$$

(7) $$\varepsilon_{c}=0.696\varepsilon_{p}$$

where *d*_0_ is the initial grain size; 

DRX volume fraction equation is:

(8) $$\varepsilon_{0.5}=3.0\times 10^{-3}d_{0}^{0.4}{\dot{\varepsilon }}^{0.086}\exp(\frac{4089}{T})$$

(9) $$X_{drex}=1-\exp\left[-0.693{\left(\frac{\varepsilon -\varepsilon_{c}}{\varepsilon_{0.5}-\varepsilon_{c}}\right)}^{1.8}\right]$$

The grain size equation is:

(10) $$d_{drex}=38d_{0}^{0.8}{\dot{\varepsilon }}^{-0.234}\exp(-\frac{42327.5}{RT})$$

where *X_drex_* is the DRX volume fraction; *ε* is the dynamic strain value; *ε*_0.5_ is the strain at a DRX volume fraction of 50%; *d_drex_* is the dynamic grain size.

### 3.2. The FE Model of the Inner Void of Steel Ball Compacted by FPM

In combination with the actual situation, the following assumptions are made during modeling.

(1) The specimen is set as a rigid plastic body since the elastic deformation is pretty small and could be neglected.
(2) The actual void shape needs to be idealized as a sphere since it is irregular and similar to spherical shape.
(3) The precondition for the void closure is that the temperature and pressure reach a certain value. Only two stages, stage of heat preservation and pressure preservation, are considered in the modeling.
(4) For easily evaluating the evolution law of the micro-structure, the initial grain size of the specimen is set to be the same.

**Figure 5:**
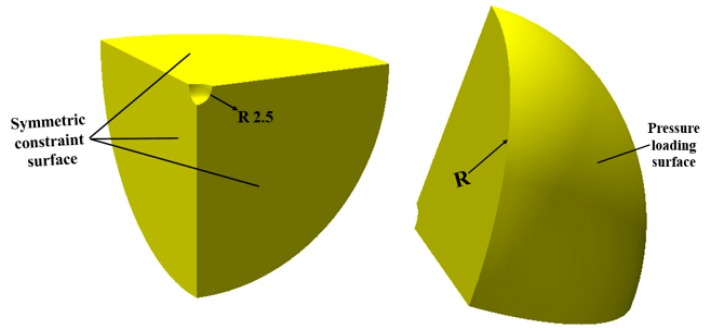
The FE model of steel ball.

The 1/8 model of the steel ball is taken for calculation in order to shorten the simulation time. A steel ball with a spherical void at the core is created in the Creo R4.0, 2015 (Formerly known as Pro/E) software. The diameter of the steel ball is 62 mm and of void is 5 mm. The 3D model is imported into the DEFORM-3D v10 software in STL (Stereolithography) format. The specimen is divided into thousands of tetrahedral element mesh, and the mesh near the void is partially refined. The boundary conditions are listed as follows: The minimum mesh unit size, initial grain size and initial temperature of the specimen is 0.2 mm, 150 μm and 1200 °C, respectively. A surface load of 150 MPa is applied to the spherical surface of the specimen and symmetric constraints are imposed on the three mutually perpendicular planes of the specimen. The heat transfer coefficient between the billet and air is 0.02 N/(s·mm·K). The FE model of the thermal-force-microstructure coupling of the internal void of the compacted steel ball is shown in Figure 5.

## 4. Void Closure Mechanism of Steel Ball Based on the FPM

### 4.1. Analysis of Void Deformation and Metal Flow Law in Steel Ball

**Figure 6:**
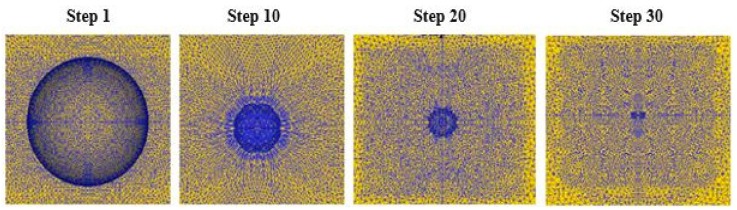
Closure law of void in steel ball.

Figure 6 shows the evolution of the internal void of the steel ball under the action of high air pressure. When the pressure increases, the void gradually becomes smaller. During the void closure, its shape remains almost spherical. This is because near the void, the three-direction compressive stress is relatively uniform so that the equivalent stress is relatively close to it. The metal flows mainly in the radial direction of the void. The closing process can be divided into two stages. The first stage is that the volume of the steel ball gradually contracts, and the second stage is the closure of the thin gap.

**Figure 7:**
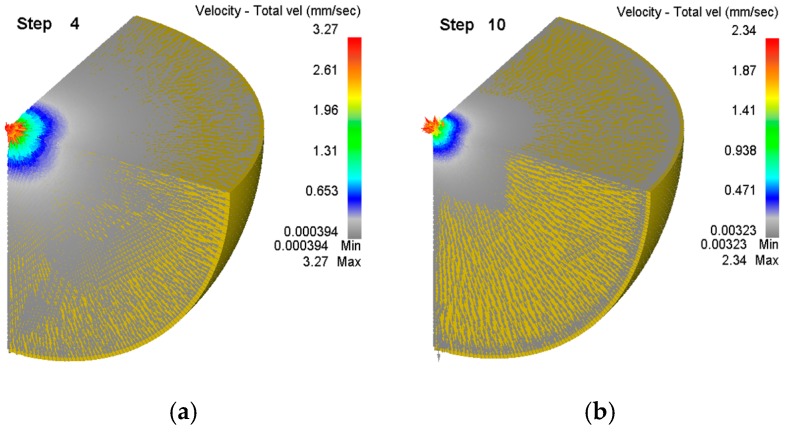
Cloud diagram of void velocity in steel ball. (**a**) The beginning of compacting the void; (**b**) At the step 10, the void is shrinking.

The metal flow rate inside the steel ball during the void closing process is shown in Figure 7.

From Figure 7, compared with the outer layer of the steel ball, the velocity line near the void is densely distributed. The degree of density of the flow line reflects the speed of the metal flow, indicating that the metal flow rate of the core of the steel ball is the fastest, and there is a tendency to flow to the void. On the outer layer of the steel ball, the flow velocity line is sparsely distributed, so the metal flows slowly along the radial direction of the steel ball. This is because the outer metal is subjected to the most uniform three-way compressive stress, which minimizes the equivalent stress.

**Figure 8:**
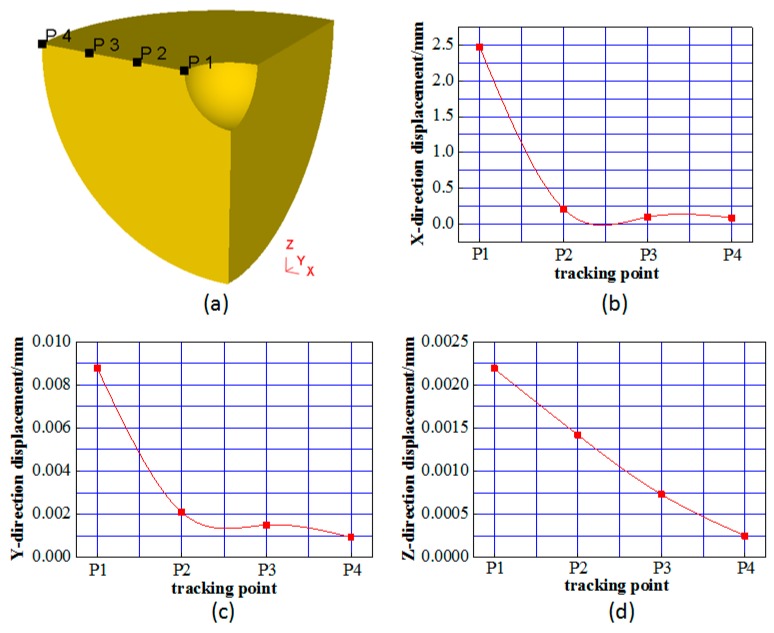
The flow law of void in steel ball. (**a**) Four tracking points on the model; (**b**) X-directional displacement of the tracking points; (**c**) Y-directional displacement of the tracking points; (**d**) Z-directional displacement of the tracking points.

The metal flow rate diagram only indicates the flow trend of the metal, which is unable to demonstrate the flow regularity of the metal. Therefore, as shown in Figure 8a, along the longitudinal section of the steel ball, four feature points are uniformly selected in the radial direction. The metal flow inside the steel ball can be obtained clearly by analyzing the three-direction displacement of each feature point. Figure 8b–d show the displacements of the three principal directions of the four feature points when the void is completely closed.

From Figure 8b, it can be seen that the closer the metal is to the surface of the steel ball, the smaller its radial movement is. The P1 point on the void contour has the largest radial displacement, which is 2.47 mm and very close to the original radius of the void. It indicates that the void is basically closed. However, it still has a small gap with a contour size of about 0.03 mm. To further close such gaps, the fine cracks can be automatically healed by the free diffusion of atoms and the dynamic recrystallization function under high temperature and high pressure conditions. In order to ensure that the fine gap can be fully healed, the steel ball needs to be insulated and dwell for a period of time because the speed of free diffusion of metal atoms is very slow. The radial displacement of the P4 point on the surface of the steel ball is 0.073 mm. According to the law that the outer metal of the steel ball has a uniform flow, the diameter of the steel ball is reduced by about 0.15 mm. The reduction in the diameter of the steel ball is much smaller than the machining allowance, and has little effect on the dimensional accuracy.

It can been concluded from the Figure 8c,d that the closer the metal is to the surface of the steel ball, the smaller the movement in the Y and Z directions. The displacement of all metals (in the steel ball) in both Y and Z directions is less than 0.01 mm. Therefore, it is considered that the metal does not flow in the Y direction and the Z direction.

In summary, during the process of void closure, the metal (inside the steel ball) mainly flows along the radial direction, causing the void change in a spherical shape. Since the diameter (of the steel ball) almost remains the same when the void is closed, there is no need to consider the influence of the flow of the outer metal on its diameter in the subsequent finishing.

### 4.2. Analysis of Internal Stress Field of Steel Ball

**Figure 9:**
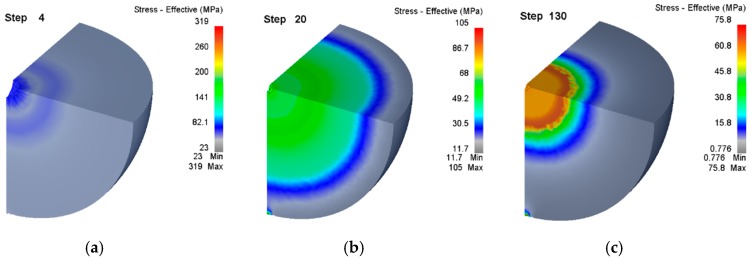
Closure law of equivalent stress distribution in the whole steel ball. (**a**) The beginning of compacting the void; (**b**) At step 20, the stress is approach to the void and its magnitude is gradually increasing; (**c**) The final step of compacting the void.

The velocity of metal flow depends on the stress. Since the steel ball is a highly symmetrical part, the internal metal always flows along the radial direction, and all the metals are in a three-way compression state, the compressive stresses of the three main directions must be taken into consideration when studying the deformation mechanism of the void. In addition, the second stage (of void deformation) is the automatic healing of the fine gap, and the healing condition is that the equivalent stress near the fine gap is greater than the yield strength of the material. Based on the factors above, we conclude the law of equivalent stress (of the steel ball) as a function of the void’s volume by analyzing the equivalent stress inside the steel ball. The equivalent stress distribution is shown in Figure 9.

As can be seen from Figure 9, from the surface to the inside, the equivalent stress shows a gradually increasing trend, because the closer to the void, the more uneven the stress of the metal in the three principal directions, and the greater the equivalent stress. As the air pressure is set and the equivalent stress on the surface of the steel ball is almost maintained at about 10 MPa with the decrease of the void volume, while the equivalent stress around the void is reduced. This is because as the void gets smaller, the smoother the contour surface of the hole is and the stress concentration decreases, thus, resulting in the decrease of the equivalent stress. After the void (of the steel ball) is completely closed, the equivalent stress in the vicinity is as high as 57.3 MPa, which is greater than the yield strength of the material of 42 MPa. At this time, the fine gap reaches the condition of automatic healing. The results above show that the smaller the void volume is, the more difficult it is to deform. If the selected air pressure and temperature are too low, the void (inside the steel ball) may not be healed. Consequently, it is critical to control air pressure and the temperature when the void inside the steel ball is repaired based on the FPM.

### 4.3. Effect of Temperature on Grain Size and DRX Inside the Steel Ball at the Final Stage of FPM

**Figure 10:**
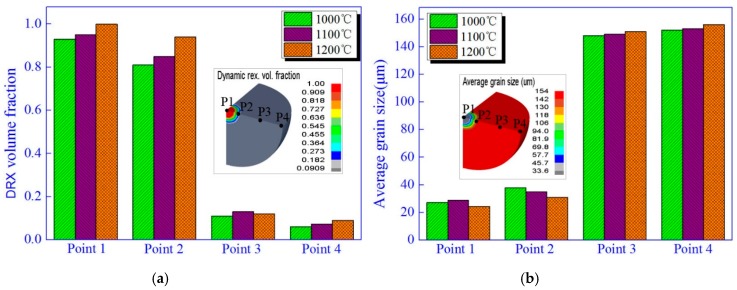
Effect of temperature on grain size and DRX in the steel ball at the final stage of FPM. (**a**) Effect of temperature on DRX; (**b**) Effect of temperature on grain size.

High temperature and plastic deformation are the main causes of DRX in the process of hot forming. FPM is performed under high temperature and air pressure, so DRX may occurs inside steel ball with macro void. Figure 10 gives the effect of temperature on grain size and DRX inside steel ball at the final stage of PFM at the air pressure of 150 MPa.

It can be found from Figure 10a the DRX volume fraction at the core and outboard position of ball are approximately 1 and 0.12, respectively, which demonstrates that the complete DRX occurs at the core of ball due to larger deformation and high temperature while almost no DRX appears at the other places of ball. Besides, the DRX volume fraction mainly presents the trend of ascend with the increase of temperature, which means that temperature in FPM is positively related with DRX. From Figure 10b, we can observe that the grain size at the core of a ball is about 24 μm, while it almost remains unchanged at the places far from the core of the ball, which can be attributed to the varied DRX volume fraction inside ball. With regard to the selected ball with macro void, a larger DRX volume fraction can not only contributes to weaken the material hardening so as to reduce the required forming force, but also promote the automatic solid welding of a needle-like gap resulting from void closure. Moreover, it is expected to obtain the fined grain inside a ball by FPM because the fined grain can improve the mechanical properties of a material. To sum up, within a proper range of temperature, the higher the temperature is, the better mechanical properties of ball might be obtained. 

### 4.4. Effect of Temperature and Air Pressure on the Stress of Steel Ball Core

**Figure 11:**
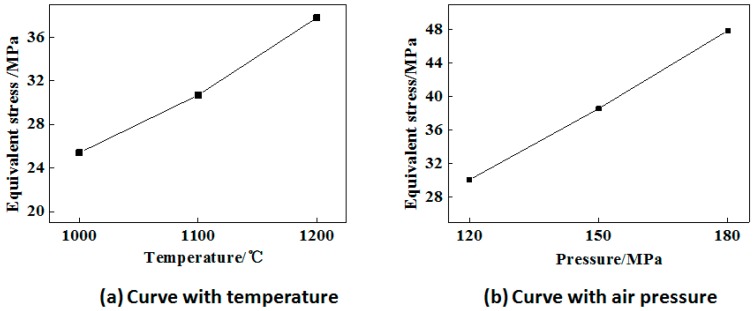
The curve of equivalent stress of steel ball core in terms of changing temperature and air pressure. (**a**) Curve with temperature; (**b**) Curve with air pressure.

The research parameters are set as follows: The diameter of the steel ball is 42 mm, with a diameter of 5 mm void in the core, and the material is 42CrMo. The temperature is selected as 1000 °C, 1100 °C, 1200 °C, and the air pressure is 150 MPa. The air pressure is selected as 120 MPa, 150 MPa, and 180 MPa, respectively, and the temperature is 1200 °C. The loading time is 20 s. At 20 s, the curves of the minimum equivalent stress (of the core in the steel ball) with temperature and air pressure are shown in Figure 11, respectively. 

According to equation 4, the void closure is affected by the equivalent stress. The greater the equivalent stress is, the easier it is to close the gap. As seen from Figure 10a, with the increasing of temperature, the equivalent stress of the steel ball’s core gradually increases. This is because, under the same air pressure condition, the internal void of the steel ball mainly changes in a spherical shape. At the same time, with the increasing of temperature, there is a higher impact on closing the void, which leads to more obvious stress concentration in the steel ball core and increasing of the equivalent stress. As seen from Figure 10b, with the increase of air pressure, the equivalent stress of the steel ball’s core gradually increases, and the influence of air pressure on the stress of the steel ball’s core is greater than that of temperature. Therefore, it is particularly important to determine the required air pressure for void closure.

## 5. Prediction of Close Pressure by Uniform Test Design Method 

Since the uniform test design method only needs to be an integral multiple of the number of times to get more data with fewer tests, the paper adopts a horizontal uniform design table, such as the Un (q)^s^, U is the abbreviation of uniform test method; *n* indicates the number of trials; q indicates the number of levels of influencing factors; s represents the number of columns in the test table. The main features of this method are as follows:

(1) For different “*n*”, a suitable design table can be constructed. The number of tests is usually selected as one or two times of the number of factors. Compared with the orthogonal test, the number of tests is obviously reduced.

(2) When the number *n* is a prime number, the number of columns is “*s* = *n* − 1”; when “*n*” is a composite number, $n={P_{1}}^{11}\cdot {P_{2}}^{12}\ldots {P_{k}}^{1k}$ where “*p_k_*” is a prime number and “1*k*” is a positive integer, the number of columns can be solved according to Equation (11):

(11) $$s=\left(1-\frac{1}{p_{1}}\right)\left(1-\frac{1}{p_{2}}\right)\ldots \left(1-\frac{1}{p_{k}}\right)$$

For the equal-level uniform test design table U_n_(n)^s^, generally, when the number of columns s is small, the orthogonal table U_n + 1_(n + 1)^s^ is constructed with n + 1, and the last row at the bottom of the table can be removed to generate U_n_(n)^s^.

In order to find out the close pressure for the internal void closure of different steel ball diameters, the two factors such as air pressure and steel ball diameter were selected as the research objects, and the experimental scheme was designed. These two parameters are divided into five levels on average, and their values are shown in Table 1:

The internal void diameter of the steel ball is 5 mm, the temperature is 1200 °C, and the material is 42CrMo. The diameter of the steel ball is in increments of 5 mm and the air pressure is in increments of 10 MPa. In order to improve the accuracy of the FE simulation results, the uniform design table U_10_(10)^10^, twice the number of simulations, is used for simulation. The simulation results as shown in Table 2 were obtained by taking the minimum equivalent stress value near the inner void of the steel ball as the index.

By transforming the data in the Table 2. into MATLAB R2014a software for quadratic polynomial fit, obtained kinds of the theoretical polynomial among the pressure, the diameter and the equivalent stress of the core of the steel ball is shown in Equation (12):

(12) $$F=F_{0}+0.442P-k_{1}d$$

where *F* is the cavity closing pressure (N/mm^2^); *F*_0_ is the barometric constant (N/mm^2^), which equals 15.107 N/mm^2^; *P* is the equivalent stress of the rolled core (N/mm^2^); *k*_1_ is the volume force coefficient (N/mm^3^) and equals 0.637 N/mm^3^; *d* is the diameter of the rolled piece (mm). 

The bearing of steel ball is 42CrMo and processed based on the FPM, and the temperature is usually fixed as 1200 °C. According to Equation (12), when $P=\sigma_{s}$, the pressure required to compact the void inside the steel balls of different diameters can be obtained. The method provides theoretical guidance for determining the process parameters when obviating internal void of the steel ball.

## 6. FPM Experiment

**Figure 12:**
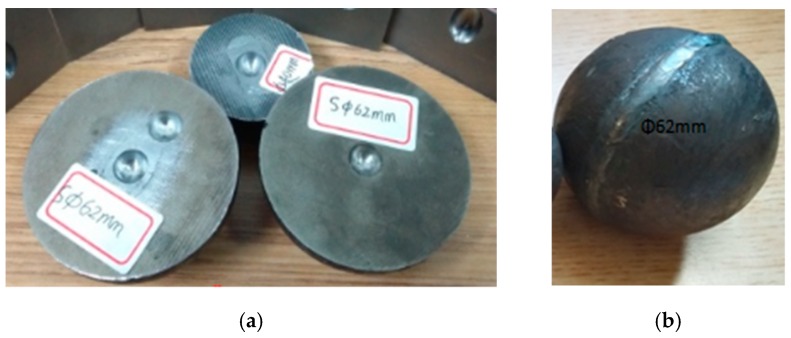
Drilling holes on the cross section of experimental specimens. (**a**) drilling; (**b**) welding together.

The steel ball is cut in the diameter direction by wire cutting, and a spherical void with a diameter of 5 mm in the radial direction in the center of the longitudinal section is intercepted. Then, the two hemispheres of the hole are closed and welded along the gap to ensure that the material properties of the weld are similar to the raw materials of the specimen, and the weld cannot remain in a small gap. The reasons why authors artificially setting up void in steel balls can be complained as follows: the real shape of void in inferior skew-rolled steel ball is irregular and similar to sphere shape, and its real size often variates from 0.1 mm to 30 mm. If using the real rolled balls to conduct the FPM experiments, there will be the following problems. (1) The real location and size of void in rolled ball are uncertain, so it is difficult to investigate the closure laws of void. (2) If there are only micro-cracks which cannot be seen with the naked eyes in rolled ball, it is difficult to verify the feasibility of eliminating defects in skew-rolled ball by FPM. Therefore, the method of artificially setting up voids in steel balls is adopted in this paper to avoid the above problems, but it is noted that the artificial void shape must be similar to the real void and its size should be larger than the real void. If the larger void in a shaft could be effectively compacted by FPM, it is ensured that the real defects in rolled balls could be eliminated. Moreover, 42CrMo with the same high temperature resistance of 1200 °C is selected as the welding material and is welded seamlessly along the weld by argon arc welding. After punching and welding, the specimen is shown in Figure 12.

**Figure 13:**
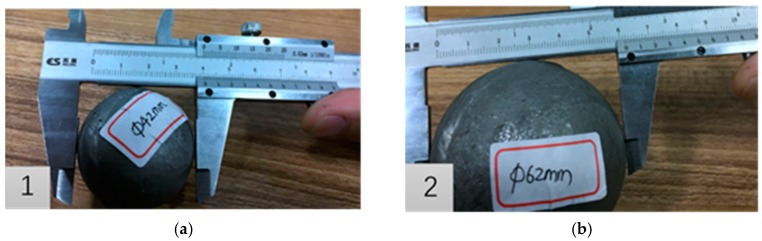
The outer dimensions measurement of specimen after the experiments. (**a**) Φ42 mm; (**b**) Φ62 mm.

**Figure 14:**
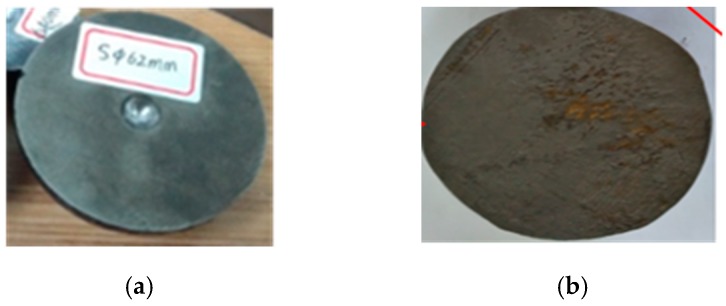
Comparable analysis of void closure in the core of specimen. (**a**) before floating pressure; (**b**) after floating pressure.

This experiment was completed in the State Key Laboratory of High Quality Special Steel Metallurgy and Preparation and High Temperature Blade Research Center of Shanghai University. The equipment (Original Design Manufacturer (ODM), Shanghai, China) that using was a large hot static press for powder metallurgy with a preset temperature of 1200 °C, a preset pressure of 150 MPa, heating for 2 h, and warming for 1 h. The shape of the specimen after the experiment is shown in Figure 13. The outer dimensions of the specimen were measured. The measurement results are shown in Table 3. It was found that the shape change of the specimen was less than 10%, and the specimen still maintained a high size precision. Finally, the specimen is cut along the weld. Figure 14 shows the change of the void on the cut surface of the specimen before and after the experiment. It can be seen that the internal void is completely closed, and there is no macroscopic discontinuity in the core metal.

**Figure 15:**
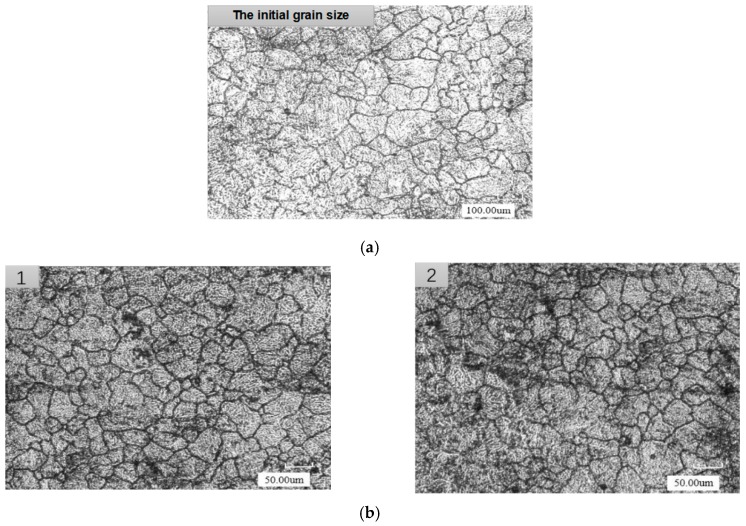
Grain size diagrams of steel ball before and after FPM. (**a**) The initial grain size; (**b**) Grain size after floating pressure method, (**1**) 10mm cube sample from Φ 42 specimen; (**2**) 10 mm cube sample from Φ 62 specimen.

In order to improve the internal quality of the steel ball, it is important to study the microstructure of the steel ball’s core after compaction. A cube piece with a side length of 10 mm was taken from the center of the cut surface of the Φ 42 and Φ 62 specimens, and the surface of the sample was polished by metallographic sample Mosaic machine. Finally, the microstructure was observed using a scanning electron microscope as shown in Figure 15, and the average grain size of each section was measured by the particle size distribution calculation software (TESCAN VEGA 3). From Figure 15, it is concluded that the average grain size of the specimens’ core decreases sharply with respect to the original 150 μm. As the diameter of the steel ball increases, the average grain size of the core increases correspondingly. Amid the same pressure and temperature, the larger the diameter is, the farther the void from the circumferential surface, the smaller the stress transmitted to the contour of the void and the smaller the strain rate of the metal near the void. All the above can lead to dynamic recrystallization activation, reduction in the dynamic recrystallization volume fraction and decrease in the degree of grain refinement. Therefore, it can be inferred that when the outer dimensions of the steel balls are the same, the smaller size of the core portion of the void, the harder it is to refine the core grain size.

## 7. Conclusions

(1) Based on the established mechanical model of FPM eliminating void in rolled steel balls, the theoretical relationships between influencing factors of void closure are obtained. Then, the metal flow behaviors, the stress distribution and the effect of process parameters on the microstructure and stress state around the void in specimens are discussed based on the established FE model. Some findings could be concluded as follows. The metal in steel ball mainly flows towards the void, and it presents a trend of increment in deformation and effective stress from the outer surface to the center of the ball. Besides, the complete DRX occurs around a void while there is almost no DRX at the other places of a ball in the circumstances of no less than 1000 °C and 150 MPa air pressure, so only the grains around void are refined. Therefore, within a certain range of temperature and air pressure, it is expected to realize complete DRX and grain refinement for obtaining better mechanical properties, so it is necessary to properly increase temperature and air pressure.

(2) Since both the effective stress and temperature at the core of the ball are the key factors of influencing the better solid welding of needle-like gaps transformed from macro voids, it is important to select proper process parameters for rolled steel balls with different diameters. Hence, the uniform design method is adopted in this work to obtain the relationships between process parameters and the effective stress at the center of balls in the case of void closures. Subsequently, the equation of the required temperature and air pressure is deduced by data linear fitting in MATLAB software, which provides theoretical guidance for the selection of process parameters.

(3) An FPM experiment is performed in a HIP (Hot Isostatic Pressing) equipment in which air pressure and temperature can reach 200 MPa and 2000 °C, respectively. Experimental results demonstrate that voids disappear and the variations of ball dimensions are less than 5% after the steel balls are subjected to the conditions of 1200 °C and 200 MPa air pressure for 2 h, which is in good agreement with the results of the numerical analysis.

## Figures and Tables

**Table 1:** Factors level table.

	Level				
Factor	1	2	3	4	5
Steel ball diameter (mm)	30	35	40	45	50
Air-pressure (MPa)	120	130	140	150	160

**Table 2:** Simulation results.

Simulation Number	Air Pressure/(MPa)	Steel Ball Diameter/(mm)	Steel Ball Core Equivalent Stress/(MPa)
1	(1) 120	(4) 45	39.17
2	(2) 130	(8) 40	46.25
3	(3) 140	(1) 30	59.03
4	(4) 150	(5) 50	50.33
5	(5) 160	(9) 45	57.05
6	(6) 120	(2) 35	48.13
7	(7) 130	(6) 30	51.17
8	(8) 140	(10) 50	45.32
9	(9) 150	(3) 40	53.11
10	(10) 160	(7) 35	65.04

**Table 3:** Comparable analysis of steel balls outward dimensions.

Result	SΦ 42 mm		SΦ 62 mm	
Object	Numerical Value	Percental	Numerical	Percental
Maximum change in diameter of steel balls	−2.17 mm	7.48%	−2.04 mm	4.86%
